# Discovery of adult T-cell leukemia

**DOI:** 10.1186/1742-4690-2-16

**Published:** 2005-03-02

**Authors:** Kiyoshi Takatsuki

**Affiliations:** 1 Department of Internal Medicine II, Kumamoto University School of Medicine, 1-1-1 Honjo, Kumamoto 860-8556, Japan

## Abstract

Adult T-cell leukemia (ATL) was first reported as a distinct clinical entity in 1977 in Japan. The predominant physical findings are skin lesions, lymphadenopathy and hepatosplenomegaly. The ATL cells are of mature T-helper phenotype and have a characteristic appearance with indented nuclei. There is striking frequent hypercalcemia with increased numbers of osteoclasts. Central to the identification of the disease is a striking geographic clustering in southwestern Japan and the isolation of human T-cell lymphotropic virus type-1 (HTLV-1) from the cell lines of patients. Worldwide epidemiological studies have been made through international collaborations. Several diseases were found to be related to HTLV-1 infection. Moreover, it was noted that an immunodeficiency state may be induced by HTLV-1 infection. In Japan, HTLV-1 carriers have been estimated to be 1.2 million, and more than 700 cases of ATL have been diagnosed each year.

## 

Adult T-cell leukemia (ATL) was described as a distinct clinical entity in 1977 in Kyoto, Japan [1,2]. The illness is manifested by presentation in adult life: frequent skin lesions, lymphadenopathy, hepatosplenomegaly, and elevated white blood cell count with abnormal lymphoid cells. The abnormal ATL cells are of mature T-helper phenotype and have a characteristic appearance with especially indented or lobulated nuclei. There is strikingly frequent hypercalcemia with increased numbers of osteoclasts. Central to the identification of the syndrome is the striking geographic clustering in southwestern Japan and the isolation of the human T-cell lymphotropic virus type-1 (HTLV-1) from the cell lines of patients with ATL.

## Background of our ATL study

It was around the 1973 that we came to recognize the existence of ATL, previously an unknown disease. I would like to give a retrospective view concerning the background of our studies on ATL.

Many clinicians in Japan probably feel that the descriptions of diseases in the literature from abroad differ from the features of the diseases observed in their practices in Japan. We can mention many examples in the field of hematology. One of these is that the incidence of chronic lymphocytic leukemia is quite low, being only 2% of all hematological malignancies. What differs is not only the incidence but also the detailed symptoms and signs of diseases. This vague feeling that an autochthonous pathology exists in Japan may be considered as key factor in the background of our study of ATL.

The second major factor is the recent progress made in basic immunology. Having an interest in immunoglobulin abnormalities, I studied multiple myeloma and related diseases in the days when even the word 'immunoglobulin' was not yet in use. The central theme of immunology at that time was to identify the structure of antibodies. Immunology has advanced rapidly, and myeloma was defined as a B-cell malignancy. Therefore, it was quite natural to enlarge the objectives of our clinical studies to all lymphoproliferative diseases.

At this stage of our research in Kyoto, joint studies with young researchers were initiated. This was the third and most important element of the background of our ATL study. I became acquainted with Drs. Takashi Uchiyama and Junji Yodoi, who are currently both working in Kyoto, during their postgraduate training. Dr. Yodoi prepared an antiserum against human thymocytes and invited us to direct our attention to T-cells.

In the course of examining patients with various lymphoproliferative disorders, we arrived at the conclusion that ATL was a disease which had not been described anywhere before. It was recognized that T-cell malignancy had a relatively high incidence among Japanese adults and that most of the patients with this disease were from Kyushu. This discovery was made from our bedside observations rather than from laboratory work. Thereafter, Dr. Uchiyama went to the National Cancer Institute, Bethesda to work with Dr. Thomas A. Waldmann and raised a monoclonal antibody, called 'Tac' antibody, which later played an important role in our ATL studies. Dr. Yodoi has developed a new field of research by identifying a novel cytokine, ATL-derived factor (ADF), which has proved to be important in redox regulation. At the end of 1981, I moved from Kyoto to Kumamoto, which is located in the middle of Kyushu. Our studies advanced remarkably there, due mainly to the efforts of excellent young co-workers including Drs. Kazunari Yamaguchi, Toshio Hattori, and Masao Matsuoka, who are now independently working in Tokyo, Sendai and Kyoto, respectively.

## Development of virology

This discovery of ATL ushered in some dramatic developments in oncology, virology and, unexpectedly, neurology and other fields of medicine. When we reported a series of 13 patients with ATL in 1976 on the occasion of the 16^th ^International Congress of Hematology in Kyoto, it was stated that 'attempt to elucidate leukemogenesis in this disease should be directed towards exploring the genetic background and a possible viral involvement'. HTLV (human T-cell leukemia virus), the pathogen of ATL, was first reported by Dr. Robert C. Gallo and his co-workers in Bethesda in 1980 and 1981. They isolated HTLV from cultured cells taken from a patient with an aggressive variant of mycosis fungoides and another with Sezary syndrome. Although both patients were said to have cutaneous T-cell lymphoma, they had some unusual features which, in retrospect, linked them to the clinical entity now called ATL.

In Japan, co-culturing of ATL cells with umbilical cord lymphocytes was first done successfully by Dr. Isao Miyoshi's group in Okayama, who obtained the cell line MT-1. Dr. Yorio Hinuma and his co-workers in Kyoto demonstrated that ATL patients have antibodies against presumed viral antigens on MT-1 cells by indirect immunofluorescence method. Subsequently, a retrovirus was isolated and called ATLV (adult T-cell leukemia virus). Since Dr. Mitsuaki Yoshida and his group in Tokyo showed that HTLV and ATLV are, in fact, identical, the term HTLV-1 (human T-cell lymphotropic virus type 1) has been commonly used.

Furthermore, the following observations have been successively reported to support the etiologic association of HTLV-1.

1. All patients with ATL have antibodies against HTLV-1.

2. The areas of high incidence of ATL patients correspond closely with those of high incidence of HTLV-1 carriers.

3. HTLV-1 immortalizes human T cells *in vitro*.

4. Monoclonal integration of HTLV-1 proviral DNA was demonstrated in ATL cells.

Thus, HTLV-1 is the first retrovirus directly associated with human malignancy.

## Diagnosis and classification of ATL

The diagnostic criteria for HTLV-1 associated ATL have been defined as follows:

1. Presence of morphologically proven lymphoid malignancy with T-cell surface antigens (These abnormal T lymphocytes include typical ATL cells and the small and mature T lymphocytes with incised or lobulated nuclei that are characteristic of chronic or smoldering type).

2. Presence of antibodies to HTLV-1 in the sera.

3. Demonstration of monoclonal integration of HTLV-1 provirus by Southern blot method.

Virological study led us to classify the patients with ATL into four clinical subtypes according to the clinical features: acute, chronic, smoldering, and lymphoma. The acute type is the prototypic ATL in which patients exhibit increased number of ATL cells, frequent skin lesions, systemic lymphadenopathy, and hepatosplenomegaly. Most of these patients are resistant to chemotherapy and have a poor prognosis. In chronic ATL, white blood cell count is mildly increased, and skin lesions, lymphadenopathy, and hepatosplenomegaly are sometimes exhibited. In the past, chronic ATL was thought to be 'chronic T-lymphocytic leukemia'. Smoldering ATL is characterized by the presence of a few ATL cells in the peripheral blood over a period of years. Frequent symptoms are skin or pulmonary lesions. Chronic and smoldering ATL often progress to acute ATL after a long period (crisis). Lymphoma-type ATL is characterized by prominent systemic lymphadenopathy, with few abnormal cells in the peripheral blood. This type has been diagnosed as nonleukemic malignant lymphoma. Later, the Lymphoma Study Group in Japan proposed a practical diagnostic criteria for classifying ATL into these four subtypes.

## Epidemiology of ATL and HTLV-1

HTLV-1 is endemic in southwestern Japan, especially Kyushu and Okinawa, in the Caribbean Islands, and in Central Africa. It has been revealed that there are HTLV-1 carriers in South America, Papua New Guinea, the Solomon Islands, South China, and other isolated populations in the world, including Ainus in Hokkaido and Aborigines in Australia. These epidemiological studies have been promoted by international collaborations, to which Drs. William A. Blattner in Bethesda, Guy de The in Paris, Kazuo Tajima in Nagoya, Shunro Sonoda in Kagoshima, and many other epidemiologists have made important contributions. Dr. Daniel Catovsky and his colleagues in London delineated the clinical features of ATL patients originating in the Caribbean islands.

## HTLV-1 related diseases

On the other hand, several diseases have been found to be related to HTLV-1 infection. Drs. Antoinne Gessain and Guy de The first reported the association of tropical spastic paraparesis (TSP) and HTLV-1, and Dr. Mitsuhiro Osame and his co-workers in Kagoshima studied features of HTLV-1-associated myelopathy (HAM/TSP) in detail. HTLV-1 uveitis was subsequently described by a study group of ophthalmologists in Kyushu. HTLV-1 may also be associated with bronchopneumonitis, arthritis, polymyositis and other inflammatory conditions. Moreover, it was noted that an immunodeficiency state may be induced by HTLV-1 infection.

## Prevention and treatment

In Japan, HTLV-1 carriers have been estimated to be 1.2 million, and more than 700 cases of ATL have been diagnosed each year. Majority of HTLV-1 transmission occurs via one of three routes, all of which require the passage of virus-infected cells. HTLV-1 carrier mothers transmit the virus to newborns mainly through breast milk. Dr. Shigeo Hino and his group in Nagasaki conducted intensive fieldwork regarding the mother-to-infant infection. Carrier mothers in Japan have been instructed to refrain from breastfeeding or modify the feeding procedures to prevent HTLV-1 infection. There is convincing evidence that HTLV-1 can be transmitted from individual to individual by sexual contact, especially males to females, and also through blood transfusions. All blood donated at the Red Cross Blood Centers in Japan has been subjected to HTLV-1 antibody testing beginning in November 1986.

Treatment of ATL is the most difficult task. Dr. Masanori Shimoyama in Tokyo has organized a multicenter study group for the chemotherapy of ATL. Many other trials have been reported: deoxycoformycin, α-interferon combined with azidothymidine, and so on. The use of humanized monoclonal antibodies against the interleukin-2 receptor has been championed by Dr. Waldmann's group. More recently, successful allogeneic bone marrow transplantation for patients with ATL has been reported from many institutions.

## Addendum

Before my retirement from Kumamoto University in 1996, I edited a book titled "Adult T-cell Leukaemia" [3]. Many of the above-mentioned investigators contributed chapters to this book. I also would like to add that Dr. Matsuoka and I wrote a chapter on ATL in a textbook of leukemia [4]. It may be pertinent here to commemorate the establishment of the International Retrovirology Association, which was announced on the occasion of the 5^th ^International Conference of Human Retrovirology held in Kumamoto on May 11–13, 1992.

A companion article by Robert C. Gallo recollects the events surrounding the discovery of the first human retrovirus, HTLV-I [5].
